# Hsp90-Dependent Assembly of the DBC2/RhoBTB2-Cullin3 E3-Ligase Complex

**DOI:** 10.1371/journal.pone.0090054

**Published:** 2014-03-07

**Authors:** Jacob R. Manjarrez, Liang Sun, Thomas Prince, Robert L. Matts

**Affiliations:** Department of Biochemistry and Molecular Biology, Oklahoma State University, Stillwater, Oklahoma, United States of America; University of Geneva, Switzerland

## Abstract

The expression of the wild-type tumor-suppressor gene DBC2 (Deleted-in-Breast Cancer 2, a.k.a RhoBTB2) is suppressed in many cancers, in addition to breast cancer. In a screen for Cdc37-associated proteins, DBC2 was identified to be a potential client protein of the 90 kDa heat shock protein (Hsp90) chaperone machine. Pull down assays of ectopically expressed DBC2 confirmed that DBC2 associated with Hsp90 and its co-chaperone components in reticulocyte lysate and MCF7 cells. Similar to other atypical Rho GTPases, DBC2 was found to have retained the capacity to bind GTP. The ability of DBC2 to bind GTP was modulated by the Hsp90 ATPase cycle, as demonstrated through the use of the Hsp90 chemical inhibitors, geldanamycin and molybdate. The binding of full length DBC2 to GTP was suppressed in the presence of geldanamycin, while it was enhanced in the presence of molybdate. Furthermore, assembly of DBC2-Cullin3-COP9 E3 ligase complexes was Hsp90-dependent. The data suggest a new paradigm for Hsp90-modulated assembly of a Cul3/DBC2 E3 ubiquitin ligase complex that may extend to other E3 ligase complexes.

## Introduction

Excluding skin cancer, breast cancer is the most common cancer, and the second leading cause of cancer deaths among women, with approximately a quarter of a million new cases of breast cancer being diagnosed annually [1]. Inherited mutations account for approximately 5–10% of breast cancer cases [2], with mutations in the BRAC1 and BRAC2 genes accounting for less than a quarter of the familial cases [3]. An additional contributing gene Deleted-in-Breast Cancer 2 (DBC2, a.k.a RhoBTB2) was identified in a region of human chromosome 8p21 that is homologously deleted in 3.5% of breast tumors [4]. Loss of this region of chromosome 8 is among the most frequent genetic defects found in prostate cancer [5], [6], and has been implicated in other common forms of cancer: ovarian [7], lung [8], [9], colorectal [9]–[11], liver [9], bladder [12], and kidney cancers [13].

In addition to these studies that have linked allelic loss in the 8p21 region to cancer, expression of DBC2 was found to be silenced in 42% of breast cancer cells or tissues [4]. Subsequent studies further found that DBC2 expression was suppressed in approximately 60% of breast cancers, 50 to 70% of lung cancers, and 75% of bladder cancers [4], [14], [15]. Loss of DBC2 expression in bladder and breast cancer was associated with aberrant methylation of the gene's promoter [14], [16], [17]. Moreover, missense mutations in the DBC2 gene were also identified in several cancers [4], [18]–[20]. Leading further support to its role as a tumor suppressor, ectopic expression of wild-type DBC2, but not its mutants, in T-47D breast cancer cells that lack DBC2 expression caused growth inhibition [4].

While DBC2 is understood to be an effective tumor suppressor gene [21], little is known about its physiological function. DBC2 is an atypical multi-domain protein containing an amino-terminal Rho domain followed by a proline-rich region, two tandem BTB domains and a conserved C-terminal domain with an uncharacterized structure [18]. The BTB domain is so named as it was originally found in Drosophila transcription factors *Bric à Brac*, *Tramtrack*, and *Broad Complex* [22]. Besides transcription, BTB-containing proteins are involved in a wide range of biological processes, including the cell cycle, the ubiquitin-proteasome system, and apoptosis [18], [22], [23].

Microarray analysis has indicated that DBC2 affects the expression of multiple gene networks regulating cell growth via cell cycle control and apoptosis, and networks related to cytoskeletal and membrane trafficking [24]. DBC2's ability to suppress cell growth has so far been biochemically linked to its ability to down-regulate cyclin D1 expression [25]. In addition, the DBC2 gene has been shown to be a direct target of the E2F1 transcription factor, whose primary function is to modulate the expression of genes involved in cell cycle progression and apoptosis [26]. Very recently, DBC2 was identified as a target gene of p53 [27]. DBC2 expression has also been demonstrated to be required for the expression of the chemokine, CXCL14 [28]. While expressed in most normal cells, CXCL14 expression is very low or absent in many cancerous cells and tumors [29]–[31], particularly those of epithelial cell origin.

DBC2s association with the cytoskeleton and membrane trafficking is supported by the observation that DBC2 functions to facilitate microtubule-mediated transport of vesicular stomatitis virus glycoprotein (VSV-G) from the endoplasmic reticulum (ER) to the Golgi apparatus [32]. Furthermore, inhibition of the migration and invasion abilities of MDA-MB-231 and MDA-MB-435 metastatic breast cancer cell lines upon ectopic overexpression of DBC2 was associated with increased protein expression of breast cancer metastasis suppressor BRMS1 and decreased phosphorylation of ezrin, a key signaling molecule that regulates cell migration and invasion [33].

The BTB-domain has structural homology with Skp1 [18], a component of Cullin1 ubiquitin ligase complexes, and directs the docking of a subset of BTB-proteins to Cullin3 (Cul3) [34]–[36]. The Cul3-bound BTB-domain containing protein functions as a substrate specific adapter for Cul3 ubiquitin ligase complexes [34]–[36]. DBC2 has been demonstrated to interact directly with Cul3, and its protein levels appear to be self-regulated through its interaction with the Cullin3 (Cul3) ubiquitin ligase complex and its ubiquitination in the absence of bound substrates [18], [37]–[39]. Tumor cell resistance to DBC2 over-expression is mediated via DBC2s rapid destruction by the 26S proteasome [40].

All the functions listed above have been proposed to occur without the binding of the Rho domain to GTP [32], [33]. In this study, we show that DBC2 is a unique client/substrate of the 90 kDa heat shock protein (Hsp90) chaperone complex. DBC2 was also found to have retained the capacity to bind GTP like the atypical Rho GTPases Rnd and RhoH. Interestingly, this GTP-binding ability of DBC2 was modulated by the Hsp90 molecular chaperone complex, and correlated with the Hsp90 ATPase cycle. Furthermore, Hsp90 was also shown to modulate the association of DBC2 with components of Cul3 E3 ubiquitin ligase complex.

## Materials and Methods

### Deletion constructs

Full length DBC2 was cloned into the Directional TOPO® pcDNA ™3.1 vector and expressed with either an N-terminal Flag- or hemagluttinin (HA)-epitope tag. Flag-tagged N-terminal deletion constructs were made similar to the full length DBC2 constructs via PCR and TOPO cloning into pcDNA3.1. C-terminal deletions of specific structural regions were constructed using site-directed mutagenesis (Stratagene) to insert stop codons at desired locations using the full-length pcDNA3.1 Flag-DBC2 as a template. All constructs were verified by DNA sequencing.

### Immunoprecipitation of HA-DBC2 from MCF7 cells

MCF7 cells were grown in 10 cm plates to 40% confluency and transfected (Roche Xtreme Gene 9) with HA-DBC2 or pcDNA3-empty vector for 16 hours. Cells were washed twice with cold PBS supplemented with 20 mM sodium molybdate and then lysed with HNET buffer (20 mM Hepes pH 7.7 with KOH, 100 mM NaCl, 2 mM EGTA, 0.5% NP-40, 5% glycerol, 20 mM sodium molybdate and Roche protease inhibitor cocktail). Lysates were centrifuged at maximum speed in a bench top centrifuge at 4°C for 15 minutes. Supernatants were then added to 40 µl of anti-HA beads (Sigma-Aldrich, A2095) and mixed at 4°C for 3 hours. Following washing of the pull-down beads 3 times with HNET buffer, adsorbed proteins were eluted with sodium dodecyl sulfate-polyacrylamide gel electrophoresis (SDS-PAGE) sample buffer. Samples were subsequently analyzed by SDS-PAGE and Western blotting for Hsp90 [OSUb2, [41]] and HA (Covance, MMS-101P)].

### In vitro coupled transcription/translation (TnT)

Flag-tagged DBC2 and each deletion construct were synthesized by couple transcription/translation (TnT) in nuclease-treated rabbit reticulocyte lysate (RRL) [42] containing [^35^S]-methionine for 30 min at 30°C with the addition of 10 µg/ml geldanamycin or dimethylsulfoxide (DMSO) at 7 min, compared to 20 mM sodium molybdate (MoO4) or deionized water at 25 min. In all cases, naïve RRL containing no template DNA was used as the control for nonspecific binding.

### Immunoprecipitation, and GMP, GTP or Ubiquitin-binding domain pull-down assays

All TnT samples were placed on ice, followed by clarification by centrifugation for 7 min at 16,000× g prior to the immuno-adsorptions or pull downs. For immuno-adsorption of Flag-tagged DBC2, 30 µl of TnT reaction mixes was added to 12.5 µl resin containing pre-conjugated anti-Flag-tag IgG (immunoglobulin G) (Sigma). For GMP/GTP pull-downs, 30 µl of TnT reaction mixes were added to 25 µl of GMP- or GTP-agarose. For ubiquitin-binding domain pull downs of ubiquitinylated DBC2, 30 µl of TnT mixes were added to 20 µl of NBR1 (KW9445), Rpn10 (KW8635) or VPS9 (KW9450) ubiquitin-binding domain-linked agarose (Enzo Life Sciences). Samples were incubated with resin for 1 h at 4°C with mixing. Samples were then washed four times with either low salt buffer containing, 10 mM PIPES (piperazine-N,N′-bis[2-ethanesulfonic acid) (pH 7.4), 100 mM NaCl, and 0.5% Tween 20 (P100T), or where noted in the figure legend once with P100T, twice with high salt buffer containing 500 mM NaCl (P500T), followed by a wash with P100T. Samples were boiled in SDS sample buffer, and analyzed by SDS-PAGE on an 8% gel, followed by electrotransfer to polyvinylidene difluoride (PVDF) membrane (BioRad), and autoradiography or Western blotting for co-adsorbed proteins. Samples quantified by scintillation counting were added to scintillation cocktail immediately following the final wash step and counted. Samples for mass spectrometry were prepared from 300 µl of TnT mixes and eluted from Flag immunoresin by 100 mM ammonium bicarbonate, 8 M urea and 4 mM EDTA. Elution was carried out at 4°C for 30 min, and then supernatant was removed and stored at −85°C until liquid chromatography 2-dimensional mass spectroscopy (LC-MS/MS) analysis.

### Competition of GTP for the binding of DBC2 to GTP-agarose

[^35^S]-DBC2 was generated by TnT in Promega T7 quick mix for 90 min, followed by centrifugation of the sample for 15 min at maximum speed in a microfuge at 4°C. The TnT mixes were split into two 200 µl aliquots which were supplemented with 23 µl 0.5 M Tris-HCl buffer (pH 8) or 0.5 M Tris HCl buffer (pH 8) containing 0.2 M GTP (final concentrations 50 mM Tris-HCl, 20 mM GTP). The GTP-agarose was pre-blocked for 1 h with 20 µg/ml bovine serum albumin, followed by 3 washes with TBS (10 mM Tris-HCl pH 8, and 150 mM NaCl). Following mixing, 30 µl aliquots of the 2 samples were mixed in triplicate with 25 µl of GTP-agarose that was suspended in 150 µl TBS or TBS containing 20 mM GTP (1∶5 dilution). Paired samples of the control and GTP-treated samples were wash immediately 4 times with 1 ml aliquots of ice cold TBS to minimize contact time of the lysate with the resin (0 min controls). The remaining triplicate samples of the control and GTP-treated lysates were incubated for 45 min at 4°C, followed by 4 washes with 1 ml aliquots of ice cold TBS. The samples were then transferred to scintillation vials and counted. The data is reported as the average of the differences between counts per min (cpm) bound at 45 min and that bound at 0 min. The experiment was carried out twice with similar results.

### Identification of DBC2-associated proteins by mass Spectrometry

Urea eluates from DBC2 pull-downs were incubated with 6 mM of reducing agent tris(2-carboxyethyl)phosphine (TCEP) for 20 minutes at room temperature. The samples then reacted with 12 mM of iodoacetamide for 15 minutes in the dark, diluted with 3 volumes of 100 mM ammonium bicarbonate pH 8.5 to reach a final urea concentration of 2 M, and digested with 8 µg/ml of sequencing grade trypsin (Promega, Cat. # V5111) overnight in dark at 37°C. The solutions were acidified with trifluoroacetic acid to a final concentration of 1%, followed by cleaned up with OMIX C18 tips (Varian, cat # A57003100). Samples were clarified by centrifugation at 20,000 rpm for 3 minutes before loading onto the mass spectrometer.

Biological samples were analyzed by LC-MS/MS on an ORBITRAP XL mass spectrometer with three or four technical replicates as indicated in the table legend using quantitative spectral counting. Centroided ion masses were extracted using the extract_msn.exe utility from Bioworks 3.3.1 and were used for database searching with Mascot v2.2.04 (Matrix Science) and X! Tandem v2007.01.01.1 (www.thegpm.org). Searches were conducted in the current IPI and SWISS Prot human database using the following search parameters: parent ion mass tolerance 10 ppm; fragment ion tolerance 0.8 Da; one missed tryptic cleavage. Gln->pyro-Glu of the N-terminus, oxidation of methionine, formylation of the N-terminus, acetylation of the N-terminus and carbamidomethylation of cysteine were specified in Mascot and X! Tandem as variable modifications. Peptide and protein identifications were validated using Scaffold v2.2.00 (Proteome Software) and the PeptideProphet algorithm. Probability thresholds were greater than 99.0% probability for protein identifications, based upon at least 2 unique peptides identified with 90.0% certainty. Proteins that contained similar peptides and could not be differentiated based on MS/MS analysis alone were grouped to satisfy the principles of parsimony. Each ID contains at least four spectral counts on average per technical replicate, and was present in all of the biological samples. The data was exported from Scaffold to Microsoft Excel for statistical analysis, with significant differences between the control and drug treated cells of p<0.05 (T-test).

### Antibodies used for western blotting

The following antibodies were used for Western bloting: Anti-Cul3 (Abcam, ab75851); Anti-DBC2 (Delta Labs, N15); and anti-CSN4/COPS4 (Abcam, ab139688). BB70 anti-Hsc70/Hsp70 and F4 anti-HOP was kindly provided by Dr. Marc Cox (UTEP). Anti-Hsp90 and anti-Cdc37 were lab made antibodies [41], [43].

## Results

### The interaction of DBC2 with the Hsp90 chaperone machine

In our ongoing studies to characterize novel proteins that interact with the Hsp90 chaperone machinery, polyclonal mouse anti-Cdc37 immobilized on agarose [44] was used to immuno-adsorb Cdc37 from K562 cell lysate in the presence of 20 mM molybdate. Following separation of Cdc37 and associated proteins by SDS-PAGE, protein bands were excised from the gel and subjected to trypsinolytic peptide mass fingerprinting. Besides the identification of p97/VCP as a Cdc37-associated protein [44], mass spectrometry and database searching (not shown) convincingly identified another protein migrating around 85 kDa, as DBC2 (a.k.a, RhoBTB2).

**Figure 1:**
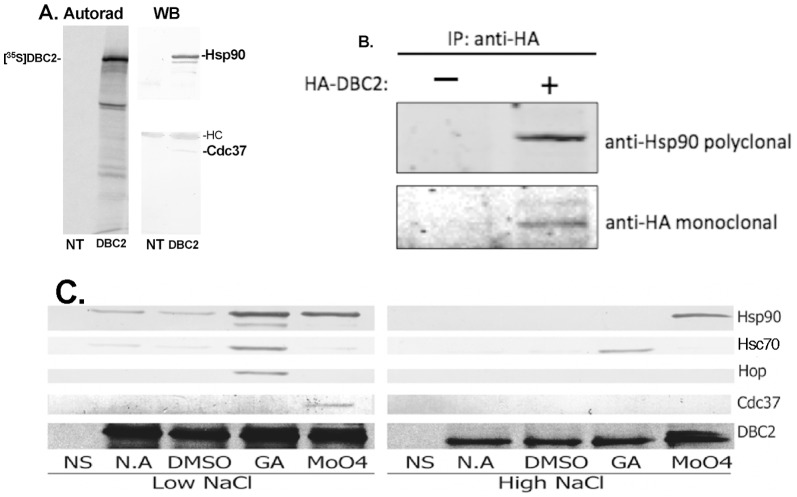
Interaction of DBC2 with the Hsp90 chaperone machine. (**A**) [^35^S]-Labeled Flag-tagged DBC2 was synthesized by TnT in RRL and immuno-adsorbed with immobilized anti-Flag antibodies in the presence of 20 mM molybdate. Samples were washed and analyzed by SDS-PAGE and Western blotting for the co-adsorption of Hsp90 and Cdc37. (**B**) MCF7 cells were transfected with pcDNA3 plasmid expressing HA-tagged DBC2 or empty vector control. After 16 h cells were lysed, and immuno-adsorbed with immobilized anti-HA-antibody. Samples were washed and analyzed by SDS-PAGE and Western blotting for the co-adsorption of Hsp90. (**C**) [^35^S]-Labeled Flag-tagged DBC2 was synthesized in the presence of geldanamycin (GA), or molybdate (MoO4), or their vehicle controls, water (no additions, NA) or DMSO, and immuno-adsorbed from RRL with immobilized anti-Flag antibodies, as described under Materials and methods. TnT RRLs containing empty vector were used as controls for non-specific binding (NS). Immuno-adsorbed samples were washed with buffer containing low (100 mM NaCl) or high salt (500 mM NaCl). Samples were analyzed by SDS-PAGE, and autoradiography (bottom panel) or Western blotting for co-adsorption of endogenous Hsp90, Hsp70, HOP and Cdc37.

To verify that DBC2 was indeed a protein that interacts with the Hsp90 chaperone machine, a cDNA I.M.A.G.E clone encoding full length human DBC2 was obtained from the ATCC and cloned into the pcDNA3.1 vector with a FLAG-tag inserted at the N-terminus of the DBC2 coding sequence. FLAG-tagged DBC2 was synthesized by coupled transcription/translation (TnT) in rabbit reticulocyte lysate, and immuno-adsorbed with anti-FLAG M2 antibody resin in the presence of 20 mM molybdate. Western blot analysis indicated that Hsp90 and Cdc37 specifically co-adsorbed with DBC2, verifying the protein as an interacting partner of the Hsp90/Cdc37 chaperone machine (Fig. 1A).

To further verify that Hsp90 interacts with DBC2, HA-tagged DBC2 was cloned into the pcDNA3 vector, which was transfected into MCF7 breast cancer cells. Cell lysates were prepared 16 h after transfection and HA-DBC2 was immuno-adsorbed with immobilized anti-HA antibody. Western blot analysis demonstrated that Hsp90 was specifically co-adsorbed with ectopically expressed HA-DBC2, but was absent from immune-adsorptions from lysates prepared from cell transfected with an empty pcDNA3 vector (Fig. 1B). Thus, the interaction of DBC2 with Hsp90 in reticulocyte lysate recapitulated the Hsp90-DBC2 interaction observed in MCF7 cells.

### The effect of Hsp90-inhibitors on the interaction of DBC2 with the Hsp90 machine

To further characterize the interaction of DBC2 with Hsp90 and components of its chaperone machine, [^35^S]-labeled Flag-tagged DBC2 was synthesized by TnT in RRL and immuno-adsorbed by the addition of M2 anti-Flag agarose in the presence or absence of Hsp90 inhibitors geldanamycin (GA) and sodium molybdate with DMSO and water as the vehicle controls. Immuno-adsorbed proteins were separated by SDS-PAGE and Western blotted for the presence of co-adsorbing components of the Hsp90 chaperone machine (Figure 1C). In the N.A. (water) and DMSO control samples, Hsp90 and Hsc70 (70 kDa heat shock cognate) were observed to co-adsorb with DBC2. While in the presence of GA, DBC2's interactions with Hsp90, Hsc70 and HOP (Hsp90-Hsp70 organizing protein) were enhanced, which is consistent with the ability of GA to stabilize client protein interactions with “intermediate” components [45] of the Hsp90 machine (Figure 1) similar to steroid hormone receptors or kinases. Addition of molybdate enhanced the interaction of DBC2 with Hsp90, and reduced its interaction with Hsc70 to below control levels, which is consistent with the ability of molybdate to stabilized “late” Hsp90 complexes that lack Hsc70 and HOP [45]. In agreement with the data shown in Figure 1A, an interaction of DBC2 with Cdc37 was only observed in the presence of molybdate.

High concentration salt washes are known to strip Hsc70 and Hsp90 complexes of their associated co-chaperones. When TnT RRL DBC2 N.A. and DMSO control pull-downs were washed with buffer containing 0.5 M NaCl, a barely perceptible interaction of DBC2 with Hsc70 was detected upon Western blotting. While in the presence of GA, high salt wash stripped Hsp90 and HOP from DBC2, with Hsc70 remaining stably bound. In contrast, in the presence of molybdate the binding of Hsp90 to DBC2 remained high after high salt wash, while Hsc70 and Cdc37 were stripped from the complex. These findings suggest that in the presence of GA, DBC2 is bound to the Hsc70 substrate binding site, as GA stabilizes “intermediate” Hsp90 complexes where substrates are bound to a hydrophobic cleft in a closed Hsp70/ADP complex that is tethered to Hsp90 through ionic interactions mediated by the TPR-domains of HOP [45]. Furthermore, the findings indicate that in the presence of molybdate, DBC2 occupies the high-affinity client binding site of Hsp90, as molybdate stabilizes “late” Hsp90 complexes where substrates have been transferred from Hsp70, which remains weakly tethered to Hsp90 through the common interaction of each protein's C-terminal EEVD motifs with the TPR-domains of HOP [45] (which is based on ionic interactions). This also indicates that the interaction of Cdc37 with DBC2 is tertiary, as it is not stabilized by molybdate in the presence of high salt [46], [47].

### The interaction of DBC2 deletions construct with the Hsp90 chaperone machine

**Figure 2:**
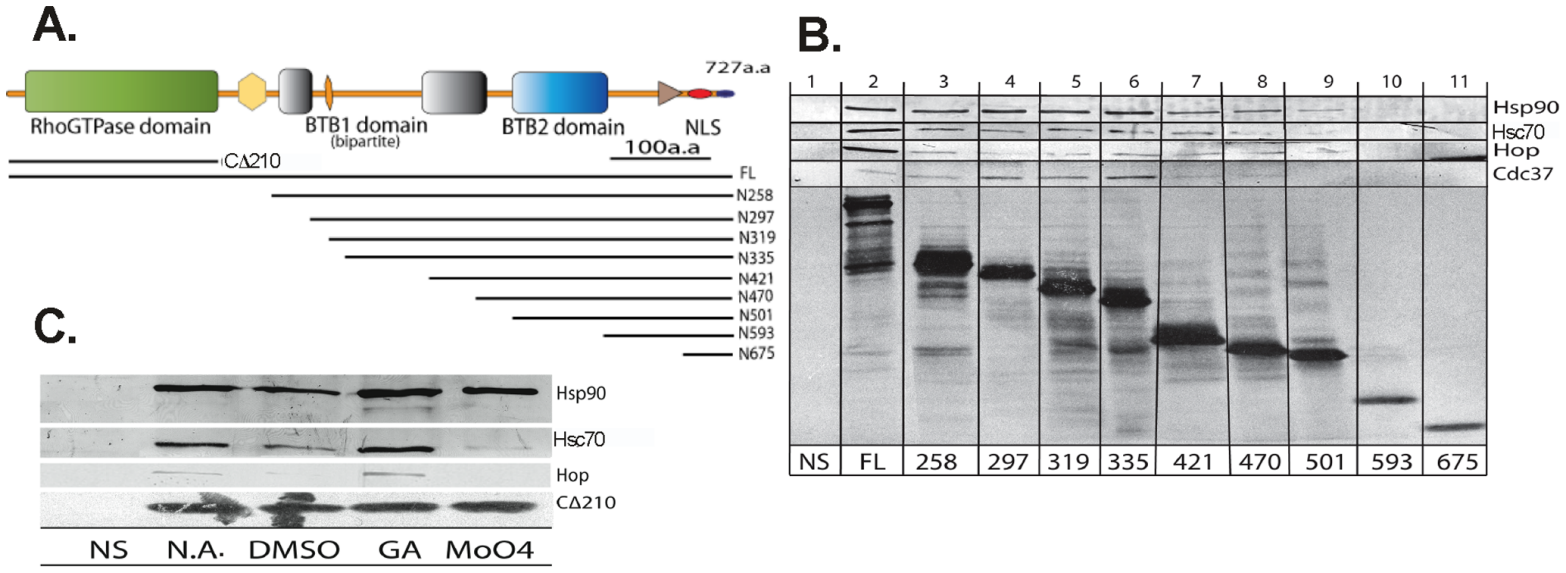
Hsp90/co-chaperone interactions with Flag-tagged DBC2 deletion constructs. (A) Summary of DBC2 deletion constructs used for the analysis. The positions of C-terminal (Ct) and N-terminal (Nt) constructs of DBC2 relative to conserved structural motifs and domains, were assigned according to SMART [67], [68], PFRAM [69] and sequence alignments. The numbers flanking each line indicates the last residue for Ct deletion and the first for Nt deletions (NCBI Accession NP_055993.2). Major domains (Rho [green], BTB1[gray] and BTB2 [light blue]) are labeled, with the Pro-rich region containing a PEST motif colored in yellow, a His-rich motif in orange, a nuclear localization signal in red, a Ser-rich element in dark blue, and a putative RING domain as the brown triangle (Supplementary Figure S1). (B) [^35^S]-Labeled Flag-tagged full-length (FL) DBC2 and DBC2 N-terminal deletion constructs, or (C) DBC2 Rho domain (CΔ210) were synthesized by TnT in the presence of geldanamycin (GA) or molybdate, or their vehicle controls water (no additions, N.A.) or DMSO, as described under Materials and methods. TnT RRLs containing empty vector were used as controls for non-specific binding (NS). Samples were then immuno-adsorbed from RRL with immobilized anti-Flag antibodies, washed with P100T, and analyzed by Western blotting for the presence of co-adsorbing proteins (Hsp90, Hsp70, HOP or Cdc37, as indicated). Bottom panels, B and C: autoradiography of [^35^S]-labeled Flag-tagged DBC2 constructs.

Previous studies have demonstrated that Hsp90 is capable of interacting with clients at more than one domain, while Cdc37 typically localizes to a single domain [46], [48]. Understanding this, deletion constructs of DBC2 were constructed to analyze the domains and/or motifs present in DBC2 that interacted with Hsp90 and components of its chaperone machine (Fig. 2). [^35^S]-Labeled Flag-tagged DBC2 and DBC2 deletion constructs were synthesized, immuno-adsorbed with anti-Flag agarose, washed with low salt buffer, and analyzed by Western blotting for co-adsorbed components of the Hsp90 chaperone machinery. Again, reactions were carried out in the presence or absence of Hsp90 inhibitors GA or molybdate, with DMSO or water as the vehicle controls. The deletions constructs are shown in Figure 2A with reference to the major DBC2 domains and sequence motifs. HOP and Hsc70, components of intermediate Hsp90 complexes interacted with the full-length DBC2, but their interactions were significantly reduced upon deletion of the N-terminal Rho domain. The interaction of the DBC2 deletion constructs with Hsp90 remained at nearly equivalent levels to that of full length DBC2 until the deletion of the first “split” BTB domain (Fig. 2B), with complete loss of Hsp90 binding being associated with the deletion of the second BTB domain. Interestingly, the deletion of the linker region between the two BTB domains resulted in a significant reduction in Hsp90 binding (Fig. 2B, N501). Enhanced binding of Cdc37 to DBC2-deletion constructs compared to full length DBC2 was observed following deletion of the Rho-domain through the first segment of the bipartite BTB domain (i.e., N297). Enhanced binding of Cdc37 was also observed for the N319 and the N335 deletion-constructs compared to full length DBC2, however this interaction was mostly lost upon deletion of the linker that connects the bipartite BTB domain.

The results above indicated that the presence of the N-terminal Rho domain was necessary for the strong interaction of DBC2 with Hsp90 intermediate complex components Hsc70 and HOP. To determine whether the Rho domain retained the capacity to interact with Hsp90, Hsc70 and HOP, [^35^S]-Flag-tagged DBC2 Rho domain (CΔ210) was synthesized, immunadsorbed to anti-Flag agarose in the presence or absence of Hsp90 inhibitors GA and sodium molybdate with DMSO and water as the vehicle controls, and analyzed for co-adsorbing proteins. Western blot analysis showed the Hsp90, Hsc70 and HOP interacted with the isolated Rho domain (CΔ210) in a manner equivalent to their interactions with full length DBC2, indicating that it is the minimal domain required for binding the components of the Hsp90 intermediate complex (Fig. 2C). Thus, full-length DBC2 and its Rho domain showed hallmarks of Hsp90 clients, exhibiting characteristic changes in their interactions with components of the Hsp90 chaperone machine in response to the Hsp90 inhibitors GA and molybdate. However, additional Hsp90-binding motifs must exist within the other domains, as interaction with Hsp90 was maintained by other DBC2 deletion constructs, albeit with significantly diminished interaction with Hsc70 and HOP and enhanced interactions with Cdc37.

### The GTP binding capacity of DBC2

**Figure 3:**
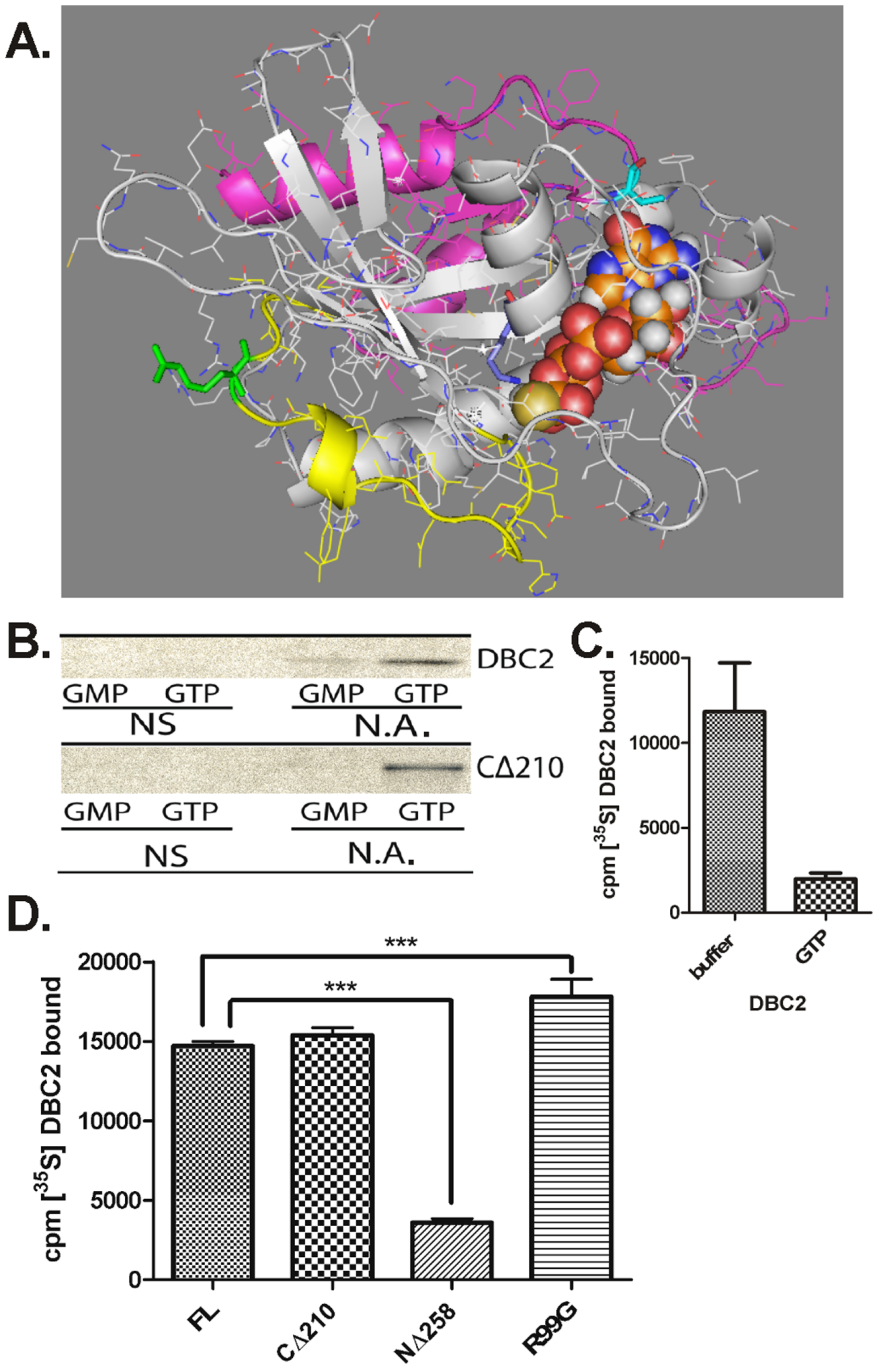
GTP binding activity of DBC2 and its Rho domain. (**A**) Homology model of the DBC2 Rho domain was built using Swiss-model and the crystal structure of Rnd1 (2CLS) as a template. The 46 residues which are deleted when DBC2's Rho-domain is truncated to a Ras domain including the loss of the G5 loop [32] are colored in magenta. The switch II region is colored yellow with the R99 residue in green. GTPgammaS is shown as spheres with V191 colored in cyan and K27 in light blue. The image was rendered by PyMol, Delano scientific. (**B**) [^35^S]- Flag-tagged DBC2 and CΔ210 were synthesized by TnT in the absence of any additions (no additions, N.A.). TnT RRLs containing empty vector were used as controls for non-specific binding (NS). [^35^S]DBC2 was pulled down from RRL with GMP or GTP-linked agarose, washed with P100T and analyzed by SDS-PAGE, and autoradiography, as described under Materials and methods. (**C**) [^35^S]-Flag-tagged DBC2 was synthesized by TnT in the absence of any additions. The samples were diluted with 5 volumes of TBS (buffer) or TBS containing GTP (GTP) to give a final concentration of 20 mM GTP. After 45 min of incubation with GTP-agarose at 4°C, the samples were washed and the amount of [^35^S]-DBC2 was determined by scintillation counting. The experiment was carried out in triplicate. (**D**) [^35^S]-Labeled wild-type DBC2, DBC2 Rho domain (CΔ210), DBC2ΔRho (NΔ258), and the DBC2/R99G (R99G) mutant were synthesized by TnT, pulled down from RRL with GTP-linked agarose, and washed with P100T as described under Materials and methods. The amount of bound [^35^S]-DBC2 constructs were quantified by scintillation counting and normalized for the number of Met present in each construct, and for [^35^S]-Met non-specifically bound to the GTP-agarose. The data represent three independent bio-replicates including three technical replicates. The (***) denotes a significant difference based on a 95% confidence interval, P<0.05.

While carrying out the domain dissection of DBC2 we aligned DBC2 with other Rho-family small G-proteins and the analysis indicated that previous work carried out on DBC2's capacity to bind GTP [32] was done with a domain construct that lacked Rho's G5 motif (Table 1; Fig. 3A), as its guanine nucleotide binding domain was truncated by 46 amino acids to a Ras domain [49]. Homology modeling of the Rho domain indicates that the truncation deletes a significant portion of a region that interacts with the guanine base in the GTP binding cleft of Rho domains (Figure 3A). The structural significance of truncating a Rho to a Ras domain (Figure 3A) lead us to re-examine the reported absence of GTP binding capacity for the DBC2 Rho domain [32]. The DBC2 Rho domain was found to have retained the necessary sequence features to bind GTP as shown by the alignment in Table 1. The small deviations from the consensus residues are shown in other atypical GTPases that have retain the ability to bind GTP even with mutations that rendered the ancestral Ras nonfunctional [49]. [^35^S]-Labeled DBC2 was generated by TnT in RRL, incubated with GTP- or GMP-agarose as the negative control. The samples were subsequently washed and assayed for bound DBC2 by SDS-PAGE and autoradiography. DBC2 was observed to specifically interact with GTP- versus GMP-agarose (Fig. 3B). While a small amount of DBC2 was observed to bind GMP-agarose, the binding was likely nonspecific as small GTPases are not know to bind GMP and quantification of the binding by scintillation counting indicated it was approximately 7% of the binding observed to GTP-agarose (11,220 cpm±1,250 versus 152,600±39,790 cpm). To further verify that the binding of DBC2 to GTP-agarose was specific, the ability of excess soluble GTP to compete with the binding of DBC2 to GTP-agarose was examined (Fig. 3C). In the presence of 20 mM GTP, the binding of [^35^S]DBC2 to GTP-agarose, as quantified by scintillation counting, was decreased by 83% (p = 0.02) compared to the control incubation. This result clearly demonstrates that DBC2 has retained its capacity to specifically bind GTP.

**Table 1:** Consensus motifs that define the structure and activity of the GTPase domain of small G-proteins.^a^

Protein	Sequence	G1 (p-loop)	G2 (switch I)	G3 (Switch II)	G4	G5
DBC2	O94844	***G***DNAV***GK***T	***PT***V	***D***TF***G***DH	CQL***D***	TSV
RhoBTB1	Q9BYz6	***G***DNAV***GK***T	***PT***V	***D***TF***G***DH	CQL***D***	TSV
Rac1	P63000	***G***DGAV***GK***T	***PT***V	***D***TA***G***QE	TKL***D***	CSA
Cdc42	P60953	***G***DGAV***GK***T	***PT***V	***D***TA***G***QE	TQL***D***	CSA
RhoA	P61585	***G***DGAC***GK***T	***PT***V	***D***TA***G***QE	NKK***D***	CSA
Rap1A	P62834	***G***SGGV***GK***S	***PT***I	***D***TA***G***TE	NKC***D***	SSA
H-Ras	P01112	***G***AGGV***GK***S	***PT***I	***D***TA***G***QE	NKC***D***	TSA
Ran	P62826	**G**DGGT***GK***T	A***T***L	***D***TA***G***QE	NKV***D***	FVA
Rnd1	Q92730	***G***DVQC***GK***T	***PT***V	***D***TS***G***SP	CKT***D***	GSA
Rnd3	P61578	***G***DSQC***GK***T	***PT***V	***D***TS***G***SP	CKS***D***	CSA
RhoU	Q7L0Q8	***G***DGAV***GK***T	***PT***A	***D***TA***G***QD	TQS***D***	CSA
RhoH	Q15669	***G***DSAV***GK***T	***PT***V	***D***TA***G***ND	TQT***D***	CSA
Consensus		***G***XXXX***GK***T	(P/A)***T***X	(***D***XX***G***)	(N/T)(K/Q)X***D***	(T/G/C)(V/S)A

a Completely conserved residues are in bold, italics.

Subsequently, we examined whether the GTP binding capacity was localized to its Rho domain. DBC2, its Rho domain (CΔ210) and DBC2ΔRho (NΔ258) were generated by TnT and assayed for their capacity to bind GTP-agarose. DBC2 and its Rho domain (CΔ210) specifically bound to the GTP-agarose at similar levels, while little binding of DBC2ΔRho (NΔ258) to GTP-agarose was observed (Fig. 3D). Thus, the GTP binding capacity of DBC2 is localized to its Rho domain. The GTP binding capacity of a mutant of the full-length DBC2 with a R99G substitution, which is in the switch II region, was also examined. The DBC2/R99G mutant, which was created serendipitously through the PCR cloning of DBC2, was observed to have increased GTP binding compared to the wild-type DBC2 and its isolated Rho domain (Fig. 3D), suggesting that the mutation may have increased the access of GTP to the binding cleft.

### The effect of Hsp90 inhibitors on GTP binding capacity of DBC2 and its Rho domain

**Figure 4:**
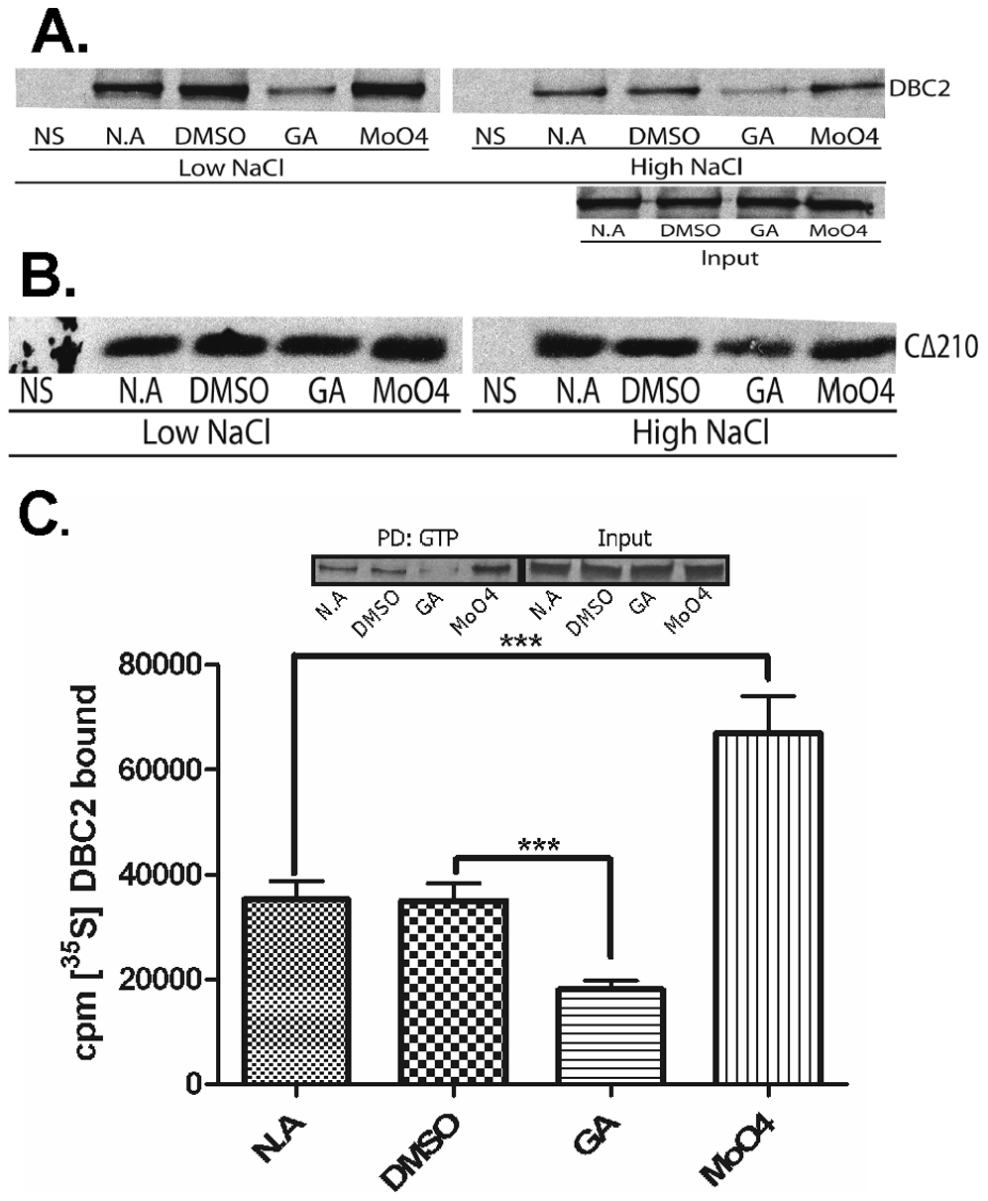
The effect of Hsp90 inhibitors on the binding of DBC2 to GTP. (**A**) [^35^S]-Labeled Flag-tagged DBC2 was synthesized in the presence geldanamycin (GA), molybdate (MoO4), or the vehicle controls water (NA) or DMSO, pulled down from RRL with GTP-agarose, washed with buffer containing low (100 mM) or high (500 mM) NaCl, and analyzed by SDS-PAGE and autoradiography, as described under Materials and methods. Naïve RRL containing no template DNA was used as the control for nonspecific binding (A and B: NS). Input: control for amounts of protein that was present in samples used for GTP-agarose pull downs. (**B**) GTP-agarose pull-downs of [^35^S]-labeled Flag-tagged DBC2 Rho domain (CΔ210) was carried out in the presence of geldanamycin, molybdate, or vehicle controls as described above in (A) for full length DBC2. (C) GTP-agarose pull downs of [^35^S]-labeled Flag-tagged DBC2 was carried out in the presence of geldanamycin, molybdate or vehicle controls as described in (A) above. The amount of bound [^35^S]-DBC2 constructs were quantified by scintillation counting and normalized for [^35^S]-Met non-specifically bound to the GTP-agarose. The data represent three independent bio-replicates including three technical replicates. The (***) denotes a significant difference based on a 95% confidence interval, P<0.05.

Since the Hsp90 chaperone machine is required for client proteins to fold into an active/functional conformation, the effect of Hsp90 inhibition on the GTP binding capacity of DBC2 was examined. The Hsp90-specific inhibitor geldanamycin significantly reduced the capacity of DBC2 to bind to GTP-agarose, while molybdate enhanced its ability to bind GTP-agarose relative to the vehicle controls (Fig. 4A and C). Washing the GTP-agarose resin with buffers containing 0.5 M NaCl (high salt) reduced the binding of DBC2 to the resin, but the observed trend of the effect of Hsp90 inhibition was still observed (Fig. 4A). Thus, Hsp90 appears to modulate the GTP binding capacity of DBC2.

The results above and those presented in Figure 3 indicate that both the DBC2 and its Rho domain bind GTP. Since DBC2's interaction with the Hsp90 chaperone machine also localized to the Rho domain, we next examined the effect of Hsp90 inhibitors on the GTP binding capacity of the Rho domain. While DBC2's Rho domain showed a small amount of reduction in its GTP binding capacity when Hsp90 was inhibited by the addition of geldanamycin (Fig. 4B), the reduction in binding was far less pronounced than that of full-length DBC2 (Fig. 4A and C). Furthermore, molybdate cause no significant increase in the GTP binding capacity of DBC2's Rho domain (Figure 4B). Again, washing the resins with buffers containing 0.5 M NaCl had only a modest effect on the binding of the Rho domain to GTP-agarose under the conditions tested (Fig. 4B). Thus, Hsp90 appears to modulate the GTP binding capacity of DBC2 primarily in the context of the full-length protein.

### Identification of DBC2-associated proteins by LC-MS/MS

**Figure 5:**
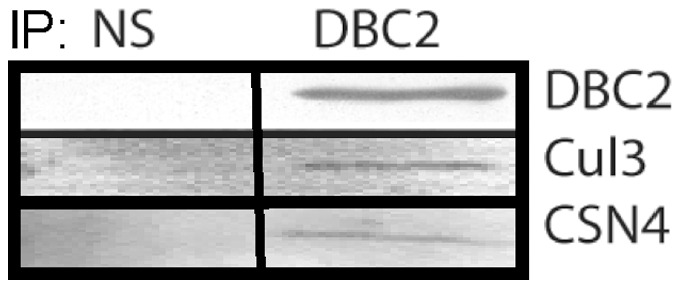
Western blots for DBC2-associated components from reticulocyte lysates identified by LC-MS/MS. Flag-tagged DBC2 was synthesized and immuno-adsorbed from RRL with anti-Flag antibody-agarose, washed with P100T, and analyzed by SDS-PAGE, and Western blotting for DBC2 and co-adsorption of Cul3 and CSN4 as described under Materials and methods.

The results presented above indicate that the capacity of DBC2 to bind GTP is modulated by the Hsp90 chaperone machine, as this capacity was decreased or increased in the presence of the Hsp90 inhibitors geldanamycin or molybdate, respectively. Using affinity purification in conjunction with LC-MS/MS, Gano and Simon elegantly demonstrated ligand-induced changes in the constituents of the Hsp90 interactome [50]. Using a similar approach, the effect of geldanamycin and molybdate on the interaction of DBC2 with proteins was examined by LC-MS/MS analysis of anti-Flag pull downs of Flag-tagged DBC2 synthesized in RRL. The effect of geldanamycin and molybdate on the association of DBC2 with components of the Hsp90 machinery were qualitatively similar to their association with DBC2 accessed by Western blotting (Table 2): geldanamycin increased the association of DBC2 with Hsp90, Hsc70 and HOP, components of Hsp90 intermediate complexes, while the Hsp90-DBC2 complexes stabilized by molybdate lacked HOP and had a decrease in bound Hsc70, consistent with molybdate's role in stabilizing late Hsp90 complexes [45]. The previously reported binding of DBC2 to Cul3 was also observed [37]–[39], and confirmed by Western blotting (Fig. 5). The presence of upward laddering of DBC2 was observed (Fig. 2B) and correlated with the ubiquitination of DBC2 in RRL as detected by pull-downs utilizing the VPS9, RPN10 and NBR1 ubiquitin-binding domains immobilized on agarose (data not shown). The binding of DBC2 to Cul3 appears to be Hsp90 dependent as it was markedly reduced in the presence of geldanamycin, but was observed in the presence of molybdate.

**Table 2:** LC-MS/MS analysis of DBC2 associated proteins in the presence or absence of Hsp90 inhibitors.^a^

			*Cumulative spectral counts*						
			*No additions* b		*Effect of Hsp90 inhibitors* c				
Protein	Gene ID	IPI Acc. No.	NS	control	NS	buffer	DMSO	GA	MoO_4_
DBC2	RhoBTB2	IPI00171950	0	152	0	95	91	123	84
Hsc70	HSPA8	IPI00003865	19	52	58	174	166	392*	129
HOP	STIP1	IPI00013894	nd	nd	0	2	0	95*	0
Hsp9œ	HSP90AA1	IPI00382470	14	12	0	28	11	153§	162§
Hsp90ß	HSP90AB1	IPI00414676	nd	nd	0	0	0	84§	70§
Cullin-3	CUL3	IPI00014312	0	138	0	42	37	5¶	29
SGN1	GPS1	IPI00156282	0	15	nd	nd	nd	nd	nd
CSN2	COPS2	IPI00743825	0	32	0	27‡	6	4	0
CSN3	COPS3	IPI00025721	0	38	0	15‡	4	0	2
CSN4	COPS4	IPI00171844	0	71	0	33	30	6†	5†
CSN5	COPS5	IPI00009958	0	24	0	14‡	0	0	1
CSN6	COPS6	IPI00163230	0	35	nd	nd	nd	nd	nd
CSN7b	COPS7B	IPI00009301	0	24	0	10‡	0	2	2
CSN8	COPS8	IPI00009480	nd	nd	0	21‡	7	0	1
RBX1	RBX1	IPI00003386	0	7	nd	nd	nd	nd	nd

a Flag-tagged DBC2 was synthesized by TnT in RRL, followed by no additions (DBC2) or treatment with geldanamycin (GA), molybdate (MoO_4_) and/or vehicle controls (buffer or DMSO). RRL containing empty vector was used as the negative control for nonspecific binding (NS). Samples were prepared and analyzed by LC-MS/MS as described under Materials and methods.

b The data for no additions represent 4 technical replicates on a single biological sample.

c The data for the Hsp90 inhibitors represent three independent bio-replicates including three technical replicates of each sample.

nd, not detected in any of that samples run for the experiment. Symbols indicate significant changes in spectral counts:

*, increase over buffer, DMSO and MoO_4_;

§ , increase over buffer and DMSO;

‡ , increase over DMSO, GA and MoO_4_;

† , decrease relative to the buffer or DMSO control; or.

¶ , decrease relative to buffer, DMSO and MoO_4_.

The LC-MS/MS analysis of DBC2-bound proteins also identified the majority of the components of the COP9 signalosome (Table 2). The COP9 signalosome is known to associate with Cul3 and function as a regulator [51]. The COP9 signalosome components have a similar pattern of interaction, and appear to associate with DBC2 upon its release from Hsp90 at the completion of its ATPase cycle, as there was a significant decrease in the association of COP9 components with DBC2 in the presence of both geldanamycin and molybdate (Table 2). The only COP9 component that seemed to persist throughout the Hsp90 cycle was CSN4. CSN4 is known to be a component of the CSN4/5/6/7 subcomplex [52], which has retained the necessary features to associate with RBX1, a known Cullin-Ring Ligase (CRL) subunit (Table 2) [53].

## Discussion

The Hsp90 chaperone machine has a plethora of client proteins that are required for almost all cellular functions and signaling pathways (current list; www.picard.ch/download/Hsp90interactors.pdf). A model for the Hsp90 ATP-driven reaction cycle was initially worked out with steroid hormone receptor (SHR) clients (reviewed in [54], [55]). The first step of the chaperone cycle involves the binding Hsc/Hsp70 and Hsp40 to SHRs that are not competent to bind hormone (early complexes [45], [54], [55]). This complex then binds HOP, which tethers the SHR-Hsc/Hsp70-Hsp40 complex to a dimer of Hsp90 (intermediate complexes). Subsequently, Hsp90 binds to ATP followed by the release of Hsc/Hsp70 and Hsp40, and the recruitment of p23 and the immunophilin FKBP52 (late complexes). The transition from intermediate to late complexes involves the dimerization of the N-terminal domains of Hsp90, and is accompanied by the activation of the SHR's hormone binding competence [54], [55]. The ATP bound to Hsp90 is then hydrolyzed to ADP, and the active SHR is released. A slightly different cycle using a somewhat different cast of Hsp90 co-chaperones (i.e., Cdc37) has been proposed for Hsp90-dependent activation of protein kinases [45], [56], [57].

The data presented in Figure 1 and 2B suggest that the processing of DBC2 by the Hsp90 chaperone machine resembles in some respects that of SHR, with Hsc/Hsp70 and HOP being associated with DBC2 present in intermediate Hsp90 complexes formed in the presence of geldanamycin, and depleted from late Hsp90 complexes formed in the presence of molybdate. Furthermore, similar to complexes formed between Hsp90 and protein kinase clients, Cdc37 was absent from Hsp90-DBC2 complexes formed in the presence of geldanamycin and present in those formed in the presence of molybdate.

**Figure 6:**
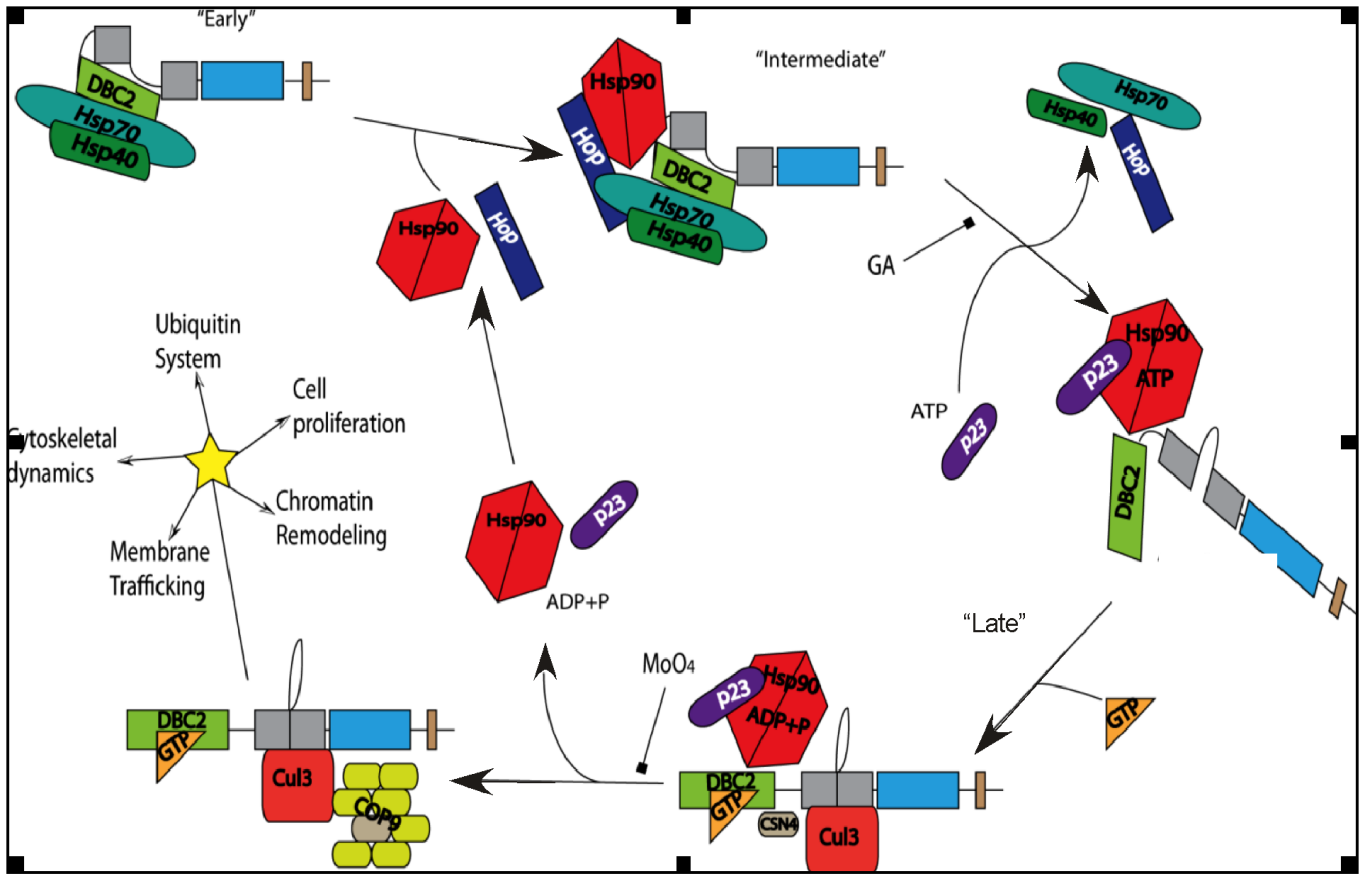
Model for the modulation of DBC2 GTP binding/activation by the Hsp90 chaperone machine. The domains of DBC2 domains are colored light green (Rho domain), gray (BTB1 domain), blue (BTB2 domain), and brown (putative RING domain). Hsp90-dependent binding of Cul3 (red) and CSN4 (brown) and other COP9 signalosome components (yellow-green) are used as example of DBC2-associating proteins. “Early”, “intermediate”, and “late” refer to complexes that are formed during Hsp90's ATPase cycle.

It has been proposed that one of the major function of the Hsp90 chaperone machine is to modulate the accessibility of ligand binding clefts [58]. The increase and decrease of GTP binding in response to inhibition of the Hsp90 ATPase cycle by molybdate and geldanamycin, respectively (Figure 4C, D), suggests a functional modulation of GTP binding similar to that by which Hsp90 prepares the hormone binding domain of the SHR to accept hormone [54] (Fig. 6). However, the lack of a significant effect of geldanamycin on the GTP binding activity of the isolated Rho domain indicates that Hsp90 does not function simply to facilitate the folding of the Rho domain into a conformation that is competent to bind GTP. The effect of geldanamycin and molybdate was only observed in the context of the full length protein. Berthold and co-workers have demonstrated that the Rho domain and first BTB domain interact, preventing the formation of functional Cul3-E3 ligase complexes [37]. Our results are consistent with a model in which Hsp90 functions to break this intramolecular interaction, freeing the Rho- and the BTB-domain to bind GTP and Cul3, respectively.

This study further examined the binding of the chaperone machine in relationship to the domain structure of DBC2. As a multi-domain protein, DBC2 appears to contain several motifs that require Hsp90-assisted folding or alignment. The interactions of DBC2 with components of the Hsp90 chaperone machinery indicate that the Hsp90/Hsc70/HOP chaperone module strongly localized to the Rho domain of DBC2. However, components of Hsp90 chaperone modules containing Cdc37 appear to interact with signaling motifs present distal to DBC2's Rho domain. Cdc37 was also demonstrated to interact with additional motifs within the DBC2 structure. While Cdc37 interacted somewhat weakly with full length DBC2, it was observed to interact with DBC2 deletion mutants extending beyond the Rho domain until deletion of the bipartite linker region of the first BTB domain. This area of the protein has several phosphorylation sites, with a strong possibility that Cdc37 participates in these kinase-catalyzed modifications [59].

Other observations suggest that the interaction of Cdc37 with DBC2 is likely indirect, as molybdate does not stabilize the association of Cdc37 with DBC2 to washing with high salt buffer [46], [47]. In addition, Cul3 has recently been shown to interact with both Hsp90 and Cdc37 [60]. Our data indicate that Cul3 binds DBC2 in the presence of molybdate, and molybdate is required for DBC2's association with Cdc37. The first BTB domain of DBC2 mediates the binding of DBC2 to Cul3 [37], [39]: an activity that is suppressed in the presence of the Rho domain [37]. Thus, deletion of first half the BTB domain likely disrupts the structure of the domain to the extent that it no longer interacts with Cul3 and its associated Cdc37.

It is presently unclear whether DBC2 is regulated by mechanisms that are similar to those that regulate the classical G-protein GTPase cycle [61]. In our LC-MS/MS analysis of proteins that co-adsorb with DBC2, we have not detected the presence of any known guanine nucleotide exchange factors (GEF), GTPase-activating proteins (GAPs), GDP disassociation inhibitors (GDIs), or the newly named GDI displacement factor (GDF) [62]. Our current hypothesis is that Hsp90 acts to facilitate a conformation change in DBC2 that makes its GTP binding site accessible to GTP, and that DBC2 like other atypical RhoGTPases lacks GTPase activity.

Atypical small GTPases have been demonstrated to function outside the framework of the classical GTPase cycle discussed above [61]. The families with characteristics that qualify them as atypical are Rnd, RhoBTB, Wrch-1/Chp, and RhoH [61]. They all have altered GTP binding/hydrolysis capabilities in common. For example, Wrch-1 (RhoU) has such a rapid nucleotide exchange rate in the absence of any substitutions within the nucleotide binding consensus sequence, that it is considered to be constitutively bound to GTP [61]. Rnd, RhoH and RhoBTB proteins have substitutions in their conserved consensus GTP binding site. However, the RhoH and Rnd proteins have been shown to remain in the GTP bound state, and to lack GTP hydrolysis activity [61]. Regulation of the Rnd G-protein is modulated through its degradation, which is required to terminate its functional activity [63]. This is the mechanism proposed for the regulation of DBC2 function, with the Cul3 ubiquitin ligase system catalyzing the auto-ubiquitination of DBC2 in the absence of bound substrates [37]–[40].

The previously reported Cul3:DBC2 interaction [37]–[40] was confirmed in this study with an expansion of the DBC2:Cul3:Ring E3 ligase complex to include its interaction with the regulatory COP9 signalosome. The COP9 signalosome binding to Cul3 is required for the activation and regulation of its E3 ligase activity, and may function to stabilize DBC2 in the absence of Cul3 target proteins, via COP9 deneddylation of Cul3 or direct COP9 mediated deubiquitination of DBC2 [64]. Regardless, the COP9 signalosome performs an obligatory function closely associated with the ubiquitin proteasome pathway, which may be linked to the ubiquitinated protein mediator complex formed between COP9, VCP and USP15, and thus it may play a potential role in the sorting of Cul3/DBC2 target proteins [65].

From our data, the following model is proposed for Hsp90 in the modulation of DBC2 that is consistent with the effects of geldanamycin and molybdate on the interaction of Hsp90 chaperone machine components with DBC2, their effect on the binding of GTP to DBC2, and DBC2's interaction with Cul3 and the COP9 signalosome. The Hsp90 chaperone machine is proposed to function as a molecular wedge that separates the DBC2 Rho domain from an intramolecular interaction with the BTB domain region, an interaction that suppresses its GTP and Cul3 binding activity. Release of DBC2 from its auto-repressed conformation would free the BTB domains for binding to Cul3 and open the Rho domain to bind GTP (Fig. 6). This model is consistent with findings that the Rho domain of DBC2/RhoBTB2 interacts with its BTB region, which maintains it in an inactive state, preventing its ubiquitination and degradation by the proteasome [37]. However, the modulation of the GTP binding and domain separation are not the only aspects of the coupling of the Hsp90 cycle to DBC2 function. The Hsp90 chaperone machine also acts as a scaffold for the assembly of DBC2 complexes. Geldanamycin-bound Hsp90 associates with DBC2 in its auto-inhibited conformation, which is lacking in GTP and Cul3 binding. Progression of the Hsp90 to the late stage of its cycle releases DBC2 from its repressed conformation stimulating the binding of GTP and DBC2's interaction with Cul3. The subsequent release of the Cul3-DBC2 complex from the Hsp90 chaperone machine allows components of the COP9 signalosome to assemble into the Cul3/DBC2 E3 ligase complex. It is not known whether neddylation of Cul3 occurs prior to its release from Hsp90, but the association of the core signalosome component CSN4 subunit with molybdate stabilized Hsp90/DBC2 complexes suggest it may be initiated while Cul3 is bound at the late stage in the Hsp90 cycle. Thus, DBC2 must transit the entire Hsp90 ATP-driven reaction cycle for it to reach its full signaling potential. Whether Hsp90 facilitates DBC2's assembly into additional non-Cul3 containing complexes, which function in other pathways, or what components are targets of Cul3/DBC2 E3 ligase complexes needs further investigation.

Several mechanistic questions remain to be answered with regard to Hsp90's role in the assembly and dynamics of the DBC2-Cul3-COP9 E3 ligase complexes. Does the Rho domain represent the substrate recognition domain of DBC2 with this function being modulated by Hsp90, or is the putative RING-finger domain (see Figure S1) near DBC2's C-terminus the substrate recognition domain? How do Hsp90 and its associated co-chaperones facilitate the interaction between DBC2 and Cul3? Does Hsp90 physically disrupt the auto-inhibitory interaction between the Rho and BTB domain to facilitate GTP binding, or does Hsp90 cause a conformational shift in GTP binding cleft of the Rho domain to allow the binding of GTP and the release of the auto-inhibitory conformation? That is, does the BTB domain physically block access of GTP to the Rho domain, or does the interaction of the BTB domain cause a conformational change in the Switch 1 and 2 regions of the Rho domain causing it to adopt a conformation incapable of binding GTP? What is the importance of Hsp90 and Cdc37's interaction with the domains of DBC2 downstream of the Rho domain? Does Hsp90 and Cdc37 possibly facilitate the phosphorylation of DBC2, a post-translational modification that may stabilize (or destabilize) the binding of DBC2 to Cul3? There is also the question of whether the Hsp90 machinery plays a role in the dynamic remodeling of Cullin-RING ubuquitin ligase networks that has been observed in other systems [66], as we have observed CAND1, a factor involved in the recycling of substrate adapters from Cullin complexes (60), to be present in immunoprecipitates of DBC2, but not reproducibly so. In conclusion, our data suggest a new paradigm for Hsp90-modulated assembly of a Cul3/DBC2 E3 ubiquitin ligase complex that may extend to other E3 ligase complexes, as many of the components of other E3 ligases, both substrate adaptors and scaffolding proteins, have been recently shown to be Hsp90 clients [60].

## Supporting Information

Figure S1 **DBC2 contains a putative RING-finger domain.**
(DOC)
