# Supplementary material for: Hsp90-Dependent Assembly of the DBC2/RhoBTB2-Cullin3 E3-Ligase Complex
Source: PLoS One. 2014 Mar 7;9(3):e90054. doi: 10.1371/journal.pone.0090054 (PMC3946479; doi:10.1371/journal.pone.0090054)
Supplement: Figure S1 — DBC2 contains a putative RING-finger domain. (DOC) [file pone.0090054.s001.doc]

**Figure S1.**  DBC2 contains a putative RING-finger domain. A Psi-blast search [1, 2] for proteins sharing a common amino acid signature with the domain following the second BTB domain of DBC2 suggests that the domain has significant sequence similarity to the N-terminal RING finger domain of CNOT4 (Expect = 1 x 10^–19^). CNOT-4 acts as an E3 ubiquitin ligase [3].

DBC2/605 MVDIDGDVLVFLELAQFHCAYQLADWCLHHICTNYNNVC----RKFPRDMKAMSPENQEY 660

M ++ D + F C YQ+ +C H I T+ N +C + +P D P +QE

CNOT4 18 MEPLEIDDVNFFPCT---CGYQICRFCWHRIRTDENGLCPACRKPYPEDPAVYKPLSQEE 74

DBC2/661 FEKHRWPPVWYLKEEDHYQRARKEREKEDYLHL 693

++ + E+ Q RK++ E+ HL

CNOT4 75 LQRIK-------NEKKQKQNERKQKITENRKHL 100

**References:**

1. Altschul SF, Madden TL, Schaffer AA, Zhang J, Zhang Z, et al. (1997) Gapped BLAST and PSI-BLAST: a new generation of protein database search programs. Nucleic Acids Res 25: 3389-3402.

2. Altschul SF, Wootton JC, Gertz EM, Agarwala R, Morgulis A, et al. (2005) Protein database searches using compositionally adjusted substitution matrices. FEBS J 272: 5101-5109.

3. Hanzawa H, de Ruwe MJ, Albert TK, van Der Vliet PC, Timmers HT, et al. (2001) The structure of the C4C4 ring finger of human NOT4 reveals features distinct from those of C3HC4 RING fingers. J Biol Chem 276: 10185-10190.
